# Heterotopic pregnancy after a single embryo transfer with successful perinatal outcome: case report and literature review

**DOI:** 10.1186/s40834-024-00266-y

**Published:** 2024-01-31

**Authors:** Wanqi Chen, Jingyi Qi

**Affiliations:** 1 The Second Affiliated Hospital of Zhengzhou University, Zhengzhou, China; 2 Luoyang Central Hospital Affiliated to Zhengzhou University, Luoyang, China

**Keywords:** Heterotopic pregnancy, Sexual intercourse, Single embryo transfer, Laparoscope

## Abstract

A heterotopic pregnancy is a rare and serious pathological pregnancy. In this paper, we report a rare case of heterotopic pregnancy and perform a literature review. A 30-year-old patient with a history of left adnexectomy presented with persistent lower abdominal pain and hemorrhagic shock after single embryo transfer. Emergency laparoscopic exploration revealed a ruptured mass in the right isthmus of the fallopian tube, for which right salpingectomy was performed. After anti-inflammatory treatment and fetal preservation, the intrauterine pregnancy progressed smoothly, and a healthy baby was delivered at 39 weeks gestation. In this case, the patient’s heterotopic pregnancy was possibly due to a natural pregnancy caused by sexual intercourse during treatment, so we recommend that sexual intercourse be avoided during transfer cycles.

## Introduction

Heterotopic pregnancy (HP) is a specific type of pathological pregnancy that involves simultaneous gestations at two sites: one a normal intrauterine pregnancy, and the other is an ectopic pregnancy [1]. HP is a rare condition in the context of natural pregnancy, with a prevalence of 1/30,000 [2], but with the introduction of ovulation stimulation and assisted reproduction techniques, the incidence of HP is increasing. Studies have shown that the incidence of this disease after ART is between 1% and 3% and that 98% of these cases involve tubal pregnancies [3]. To reduce the incidence of high-risk pregnancies after ART, such as multiple pregnancies and ectopic pregnancies, single-embryo transfer (SET) has been widely accepted worldwide.

However, in women who undergo single embryo transfer, early HP monitoring can be overlooked. Here, we present a case of HP after SET, which resulted in the delivery of a healthy full-term infant after laparoscopic surgery.

## Case report

A 29-year-old female patient with “primary infertility, a left ectopic ovarian cyst, and bilateral fallopian tube obstruction” was admitted to the Reproductive Medicine Department of our hospital for “in vitro fertilization-embryo transfer”. After 14 days of ovulation stimulation, 15 eggs and 7 available blastocysts were obtained. Clinical pregnancy was confirmed after the fresh transfer of a blastocyst. At 18 weeks of pregnancy, the patient underwent open surgery at a local hospital to remove the left fallopian tube and part of the ovary due to the “rupture of an ectopic cyst on the left ovary.” Spontaneous abortion occurred after surgery.

One year later, the patient underwent endometrial transformation after ovulation following monitoring of follicle development during a natural cycle, and one frozen embryo was transferred. At 14 and 18 days after transfer, the blood ß-HCG levels were 1307.57 miu/ml and 9331 miu/ml, respectively.

At 34 days after transfer, the patient had persistent lower abdominal pain of no apparent cause. Her body temperature was 36.5 °C, her pulse was 70 beats/min, her respiration was 20 times/min, and her blood pressure was 80/50 mmHg. Ultrasonography indicated an early intrauterine pregnancy and pelvic effusion. After two-channel fluid correction of shock, reexamination via color ultrasound revealed: early intrauterine pregnancy, the uneven echo mass in the right adnexal area about 3.1 × 3.1 cm, and a deep effusion of about 2 cm behind the uterus.Emergency laparoscopy was performed the same day. During the operation, a large amount of blood and blood clots were found in the pelvis and abdominal cavity, with a volume of approximately 2000 mL. The right isthmus of the fallopian tube was enlarged by 5 × 4.3 cm. A ruptured pregnancy sac of 1.5 cm in diameter was observed, and a clot was attached to the surface. Then, laparoscopic right salpingectomy was performed. Postoperative ultrasonography confirmed intrauterine embryo survival.

In postoperative communication, the patient stated that sexual intercourse had occurred during the transfer cycle. Postoperative pathology indicated a pregnancy in the right isthmus of the fallopian tube with typical villi. Based on the clinical presentation, auxiliary examination, and intraoperative findings, the patient was diagnosed with HP.

**Fig. 1:**
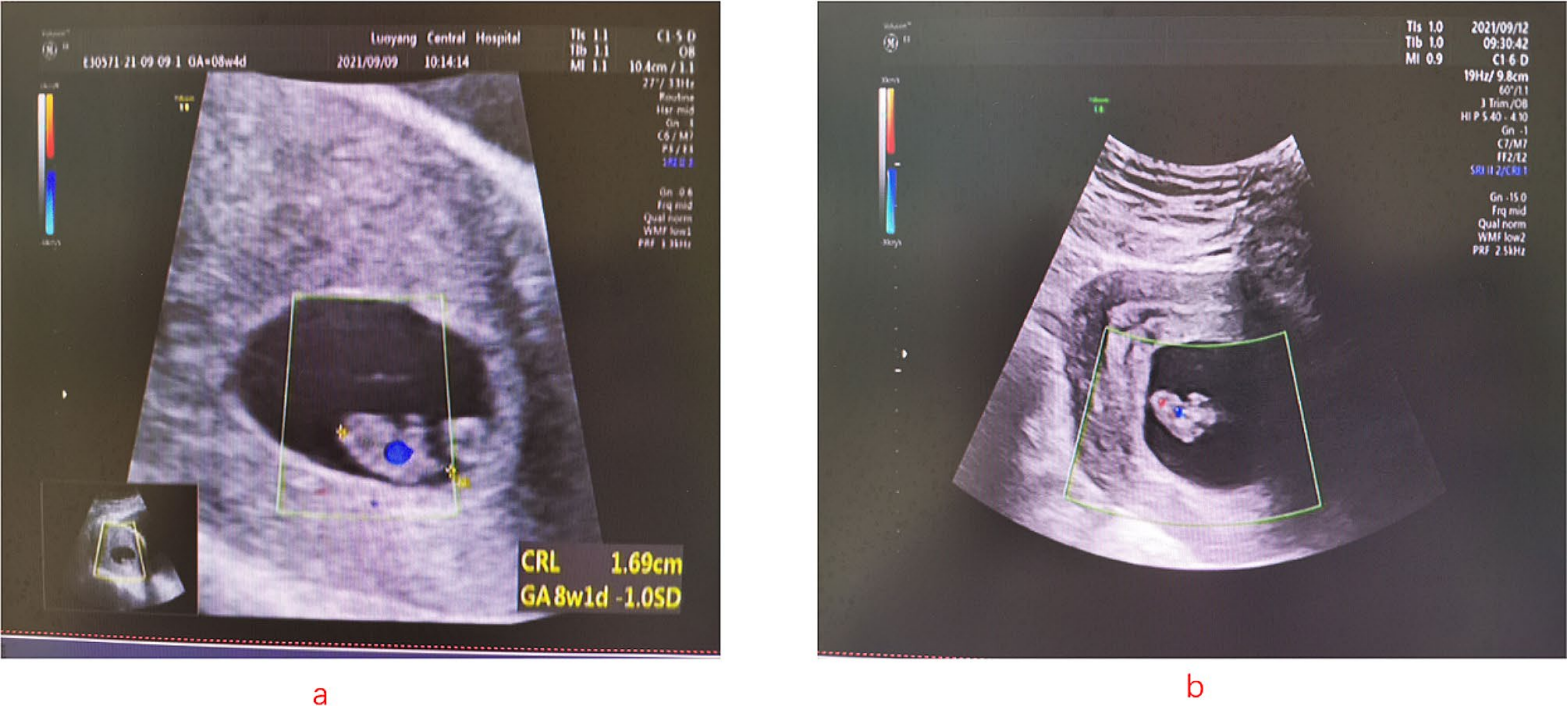
Figure (a) shows the ultrasound on the second postoperative day. Figure (b) shows the ultrasound on the fifth postoperative day. Both indicate intrauterine pregnancy

**Fig. 2:**
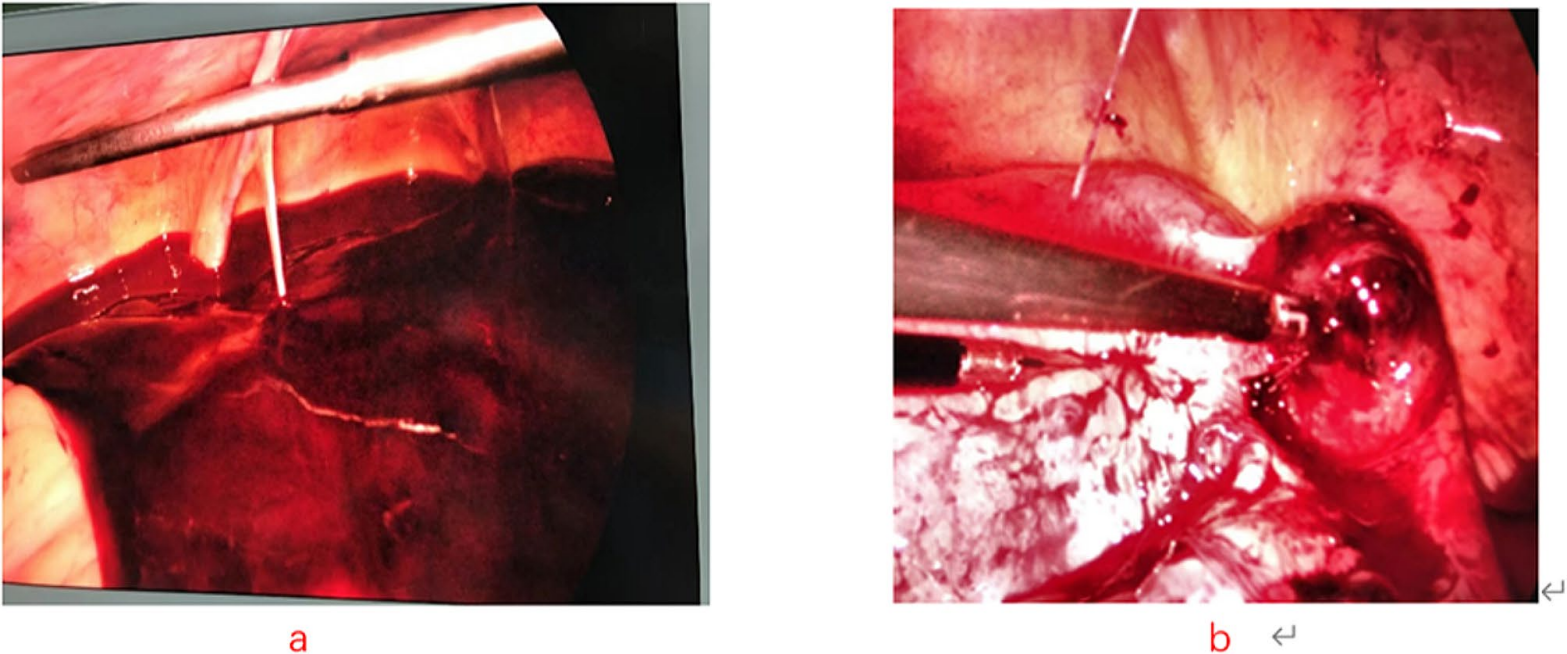
Finger (a) shows the patient has a maasive abdominal hemorrhage. Finger (b) shows the gestation sac on right tubal isthmus

A cesarean section was performed at 39 weeks, and a healthy newborn was delivered (Figs. 1 and 2).

## Discussion

We performed a literature search in PubMed using the terms “single embryo transfer” and “heterotopic pregnancy” and found four cases of HP after SET [4–7]. Regardless of treatment or outcome, we registered reported data on the clinical presentation, risk factors, management and outcomes. Table 1 summarizes the information of patients with HP after SET.

**Table 1:** Review of the cases of HP after SET

	Case 1 [4]	Case 2 [5]	Case 3 [6]	Case 4 [7]	our case
Age	34	39	40	33	30
Gestational week of intrauterine pregnancy	11 weeks	7 weeks and 5 days	5 weeks and 2days	6 weeks	7 weeks and 6 days
Extrauterine pregnancy sac size	Not described	51 *40 mm	19mm	Not described	50*43mm
Gestational week of extrauterine pregnancy(Based on the size of the ectopic pregnancy sac)	Not described	5weeks	3weeks	Not described	5weeks
Past medical history	diabetes, ovarian hyperstimulation syndrome	Twice cesarean section	primary infertility,pelvic inflammatory disease	Left salpingectomy for EP, hyperthyroidism	Left adnexectomy, primary infertility
ART method	Fresh ET	FET	FET	FET	FET
Sexual intercourse	(+)	(+)	(+)	(+)	(+)
Ultrasonographic findings	A conspicuous amount of intra-abdominal fluid with several blood clots were present. The ovaries were still increased in volume	A mass with a diameter of 51 x 40 mm was detected at the right adnexa	A dilated tubular structure of 1.9-cm-thickness in the right lower quadrant of her abdomen	Ascites	An uneven echo mass in the right adnexal area about 3.1 x 3.1cm, and a deep effusion of about 2cm behind the uterus.
Clinical symptoms	Hypotension, abdominal pain, weak peripheral artery pulsation, pale skin and cold extremities	Lower abdominal pain and vaginal bleeding	Lower quadrant colicky pain	Abdominal pain	Hypotension, abdominal pain, weak peripheral artery pulsation, pale skin and cold extremities
Treament	Laparoscopic right salpingectomy	Laparoscopic right salpingectomy	Laparoscopic right salpingectomy	Laparoscopic left salpingectomy and interstitial wedge resection	Laparoscopic right salpingectomy
Outcome	Pregnancy termination at 23 weeks of gestation because of fetal anomaly	Intrauterine fetal death at 19 weeks of gestation	Transvaginal delivery at 39 weeks of gestation	Cesarean section at 36 weeks of gestation	Cesarean section at 39 weeks of gestation

Reviewing the medical histories of the five patients, we found that one patient had pelvic inflammatory disease and two patients had a history of ectopic pregnancy and salpingectomy, which are all risk factors for ectopic pregnancy. We also collected data from 5 patients with twin pregnancies after single embryo transfer [8–11], and reviewed their related medical history. Of the five patients, one patient had a history of ovectomy, and two patients had blocked fallopian tubes. These factors are not risk factors for HP after SET. We found that patients with both post-SET HP and twin pregnancies had sexual intercourse during the transfer cycle. Therefore, an important cause of HP after SET may be unprotected sexual intercourse.

Regarding the symptoms of these patients, all five patients had abdominal pain as the first symptom, and two patients had shock symptoms. Ultrasonography revealed abnormalities in the accessory area. This indicated that the ectopic pregnancy sac had ruptured, which has irreversible effects on the pregnant woman and the embryo in the uterus. To diagnose HP early and avoid severe bleeding and maternal death, clinicians should inform patients about careful and frequent follow-up with weekly ultrasounds during early pregnancy. Transvaginal ultrasound should be selected as far as possible. Studies have shown that the sensitivity of transvaginal ultrasound can reach 96% and the specificity can reach 93% for the diagnosis of HP [12].When examining the intrauterine pregnancy sac, the ultrasound physician should also pay attention to indirect signs of pregnancy, such as a mass in the accessory area and fluid accumulation in the pelvic cavity. Some scholars have formulated the following criteria to facilitate timely preoperative diagnosis [13]:(1) uterine enlargement conforming to the month of the last menstrual period; (2) enlargement of the uterus accompanied by the luteal development of both ovaries; (3) no retraction bleeding during ectopic pregnancy operation and persisting pregnancy manifestations; and (4) an intrauterine pregnancy with unexplained abdominal bleeding. When a patient is suspected to have an ectopic pregnancy sac, further examination may be performed in conjunction with MRI. When abdominal bleeding, shock or other emergencies occur, laparoscopic exploration is the best examination method  1–2.

In terms of the choice of treatment, laparoscopic salpingectomy was performed in all five of the above patients. Researches show that salpingectomy is the first choice for women with HP after IVF-ET [14]. Moreover, this approach can greatly shorten the operation time, reduce intraoperative stimulation of the uterus, and reduce the probability of another ectopic pregnancy [15]. Clinicians should avoid using intrauterine operators during laparoscopic surgery, avoid touching the enlarged pregnant uterus after entering the abdominal cavity, and use bipolar electric coagulation equipment for electrical coagulation in the shortest amount of time to avoid the adverse effects of electric heating energy on the intrauterine pregnancy. Warm saline, rather than cold saline, should be used when irrigating the surgical field to reduce stimulation of the uterus so as not to induce contractions.

Women with HP show no clinical symptoms; early detection and an unruptured ectopic pregnancy sac are important when determining medical treatment methods. Because patients desire to preserve intrauterine pregnancies, fetal teratogenic drugs should be avoided during drug selection and should be used locally at low doses. The method of local administration is to perform extrauterine gestational sac puncture under the guidance of ultrasound. To improve the efficacy and safety of methotrexate treatment, the indications for methotrexate are a serum β-hCG concentration less than 1500 IU/L, an undetectable fetal heart rate, and a pregnancy follicle size not exceeding 35 mm [16]. A single dose of 50 mg of MTX was reported by Sijanovic et al. [17] Three weeks after MTX injection, the ectopic pregnancy subsided, and the intrauterine pregnancy was still present. Recent studies have shown that [18]: local injections are more suitable for treating ectopic pregnancies in C-section scars or in the cervix, the prevalence of ectopic tubal pregnancies is low, and the newer drugs letrozole and gefitinib may improve treatment efficacy; however, there is insufficient evidence proving the efficacy of these new drugs.

If a patient has no clinical symptoms and the extrauterine fetus has died, expectant treatment may be considered. The advantage of this approach is that it avoids the side effects of surgery and drugs. If expectant treatment is chosen, the frequency of follow-up should increase so that abnormalities can be detected at any time. In 2022, Ayyash et al. [19] reported a case in which expectant treatment was successful and cesarean section delivery occurred at 38 weeks gestation.

In terms of pregnancy outcomes, among the above five patients, one patient chose to induce labor due to fetal malformation, one patient experienced intrauterine fetal death without an obvious cause, and the other three patients had healthy intrauterine pregnancies. The outcome of intrauterine pregnancies is usually good. Transvaginal ultrasound and early surgical intervention for HP is key for preventing adverse outcomes and saving an intrauterine pregnancy [20]. However, we need to inform patients about the perioperative risks of a live birth and follow them up regularly after successful surgery.

Although previous reports have shown that sexual intercourse can improve early embryo survival [21], this case report and literature review revealed that sexual intercourse during the treatment cycle can lead to HP and a high risk of twin pregnancy; therefore, we recommend that sexual intercourse be avoided or contraception be used during transfer cycles.

## Data Availability

No datasets were generated or analysed during the current study.

## Declarations

This case report has been written with the patient’s consent.
